# COVID-19 as a Risk Factor for Peri-Implant Disease: A Prospective Clinical Study

**DOI:** 10.7759/cureus.92856

**Published:** 2025-09-21

**Authors:** Naresh Vabanaboyina, Chiramana Sandeep, Thanjavuri Krishna, Shahista Afreen, Vijay B Kumar, Amirneni Srihita, Seema Gupta

**Affiliations:** 1 Department of Prosthodontics, Sibar Institute of Dental Sciences, Guntur, IND; 2 Department of Oral and Maxillofacial Surgery, Sibar Institute of Dental Sciences, Guntur, IND; 3 Department of Conservative Dentistry and Endodontics, Sibar Institute of Dental Sciences, Guntur, IND; 4 Department of Orthodontics, Kothiwal Dental College and Research Centre, Moradabad, IND

**Keywords:** covid-19, dental implants, osseointegration, soft tissue, stability

## Abstract

Introduction: The systemic effects of coronavirus disease 2019 (COVID-19) may influence bone metabolism, potentially impacting the success of dental implants. This study investigated the effects of prior COVID-19 infection on hard- and soft-tissue outcomes following dental implant placement with the aim of providing insights for optimizing treatment planning in prosthetic dentistry.

Materials and methods: A prospective comparative study was conducted at the Department of Prosthodontics, Sibar Institute of Dental Sciences, Guntur, India, between May 2022 and December 2023. Twenty partially edentulous patients (aged 20-60 years) were divided into two groups: Group A (n = 10, COVID-19 history) and Group B (n = 10, non-COVID-19 history). The inclusion criteria were mandibular posterior edentulism and sufficient bone dimensions confirmed using cone-beam computed tomography (CBCT). Group A required confirmation of a prior COVID-19 infection via reverse transcription polymerase chain reaction (RT-PCR). The exclusion criteria were uncontrolled systemic diseases and smoking. Dental implants (AlphaBio ICE, Petach Tikva, Israel) were placed using surgical stents and preoperative antibiotics. The parameters assessed at baseline and at three and six months included mucosal thickness, bleeding on probing (BOP), implant stability (Osstell ISQ; Göteborg, Sweden), and marginal bone loss (via intraoral periapical (IOPA) radiographs). The data were subjected to statistical analyses.

Results: Both groups had comparable demographics (mean age: 34.10 ± 4.15 years). Non-COVID-19 patients showed implant stability (p = 0.634), significant marginal bone loss (p = 0.001), and mucosal thinning at six months (p = 0.001). COVID-19 patients exhibited marginally improved implant stability from three to six months (p = 0.06), significant bone loss (p < 0.001), and progressive mucosal thinning (p < 0.001). Between-group comparisons indicated lower implant stability (p = 0.02) and reduced mucosal thickness (p = 0.01) in COVID-19 patients at three months, with marginally greater bone loss at six months (p = 0.05).

Conclusion: A prior COVID-19 infection may contribute to delayed osseointegration, increased bone loss, and altered soft tissue healing, necessitating tailored treatment strategies and vigilant monitoring in implant dentistry.

## Introduction

Dental implants have revolutionized prosthetic dentistry by offering a reliable and aesthetically pleasing solution for replacing missing teeth. Unlike traditional methods, such as fixed or removable partial dentures, dental implants provide a restoration that closely mimics the function, comfort, and appearance of the natural tooth without compromising adjacent healthy teeth [1]. Defined as alloplastic structures implanted into oral tissues beneath the mucosa, periosteum, or bone, dental implants serve as a stable foundation for fixed or removable prostheses. Their predictable success in treating complete and partial edentulism has made them an integral modality in modern dentistry, particularly for single-tooth replacements where preserving neighboring teeth is a priority [1,2].

The global outbreak of coronavirus disease 2019 (COVID-19), caused by severe acute respiratory syndrome coronavirus-2 (SARS-CoV-2), has introduced unprecedented challenges to healthcare, including dentistry [3]. Beyond its well-documented pulmonary complications, such as pneumonia and acute respiratory failure, COVID-19 has been implicated in systemic effects, including alterations in bone metabolism [4]. The virus interacts with the angiotensin converting enzyme-2 (ACE-2) receptor, which is expressed not only in respiratory epithelial cells but also in bone tissue by osteoblasts and osteoclasts. This interaction disrupts the delicate balance between bone apposition and resorption, favoring osteoclast activity and leading to bone resorption [4,5]. Consequently, patients with a history of COVID-19 may experience reduced bone mineral density and low serum calcium levels, potentially compromising the osseointegration of dental implants [6].

Osseointegration, the direct structural and functional connection between the living bone and the implant surface, is critical for the long-term success of dental implants. However, the inflammatory cytokine storm induced by COVID-19 and its impact on the ACE-2 pathway may exacerbate bone resorption, potentially leading to adverse hard- and soft-tissue changes after implant placement [6]. These changes include crestal bone loss, altered implant stability, variations in probing depth, bleeding on probing (BOP), mucosal thickness alterations, and papilla changes [6,7]. While these outcomes are expected to some extent in all implant patients, the extent and progression of these changes in individuals with a history of COVID-19 remain poorly understood owing to limited long-term data.

Given the potential influence of COVID-19 on bone metabolism and its implications for dental implant success, there is a pressing need to investigate how these systemic effects translate into improved clinical outcomes in implant dentistry. This study aimed to address this gap by evaluating and comparing hard and soft tissue changes at various time intervals following dental implant placement in patients with and without a history of COVID-19. By examining parameters such as mucosal thickness, BOP, implant stability, and marginal bone loss, this study sought to provide insight into the impact of COVID-19 on dental implant outcomes. Such knowledge is essential for clinicians to optimize treatment planning and predict long-term success in this unique patient population, ultimately enhancing the quality of care in prosthetic dentistry.

## Materials and methods

This prospective comparative study was conducted at the Department of Prosthodontics, Sibar Institute of Dental Sciences, Guntur, India, over a period of 18 months from May 2022 to December 2023. Ethical approval was obtained from the Institutional Ethical Committee (IEC) prior to the commencement of the study (Pr.78/IEC/SIBAR/2022), and all procedures adhered to the principles outlined in the Declaration of Helsinki (2013 revision) [8]. Written informed consent was obtained from all patients after a detailed explanation of the study’s objectives, procedures, potential risks, and benefits.

Power analysis was performed using GPower 3.1.9.7 software (Heinrich Heine University, Düsseldorf, Germany) with an independent t-test. With an effect size of 1.15, an alpha level of 0.05, and a power of 0.80 across two groups, the analysis determined that a total sample size of 20 (10 per group) was sufficient to detect significant differences. The effect size estimation was derived from prior research on marginal bone loss in COVID-19 and non-COVID-19 patients [9].

Twenty partially edentulous patients aged 20-60 years were selected based on stringent eligibility criteria. Inclusion criteria were mandibular posterior partial edentulism, sufficient bone height and width for implant placement as confirmed by cone-beam computed tomography (CBCT), willingness to provide informed consent, and, for the COVID-19 group, availability of real-time reverse transcription-polymerase chain reaction (RT-PCR) reports confirming prior COVID-19 infection. The exclusion criteria were uncontrolled systemic diseases (such as diabetes mellitus and hypertension), recent radiation therapy to the head and neck region, habitual substance abuse (such as smoking, alcohol, or drug use), or any contraindication to implant surgery.

Twenty patients were divided into two groups: Group A consisted of 10 (50%) patients with a COVID-19 history, and Group B consisted of 10 (50%) patients with a non-COVID-19 history, based on their history of COVID-19 infection. A thorough case history, including medical and dental records, was collected to rule out contraindications. For Group A, RT-PCR reports were reviewed to confirm prior COVID-19 infection. Routine blood investigations, including complete blood count, hemoglobin, and blood glucose levels, were performed preoperatively in all patients. Diagnostic impressions were made using irreversible hydrocolloid impression material (Algitex, DPI, Uttarakhand, India), and type III gypsum products (Kalstone, Kalabhai, Mumbai, India) were poured to obtain diagnostic casts. Preoperative CBCT scans (Carestream, Rochester, NY, USA) were performed to assess bone dimensions (length, width, and height) at the implant site. Patients were prescribed prophylactic antibiotics (amoxicillin 500 mg, three times daily; Alkem Laboratories, Mumbai, India) one day prior to surgery.

Surgical stents were fabricated to guide implant placement. Reference lines were drawn on the diagnostic cast: Line A along the crestal center of the edentulous ridge, extending from the central fossa of adjacent teeth, and Line B perpendicular to Line A buccal to lingual at the midpoint of the edentulous site. The intersection of Lines A and B indicated the dental implant position. An acrylic tooth (Premadent, Super Dental Products, Delhi, India) was placed in the edentulous region, and a wax-up was performed using modeling wax (Maarc, Mumbai, India). A separating medium was applied, and a surgical stent was fabricated using an auto-polymerizing acrylic resin (DPI, Uttarakhand, India). A 2 mm hole was drilled at the intersection point using a No. 6 round tungsten carbide bur with a laboratory micromotor (Marathon 4, Japan) to guide the pilot drill.

On the day of surgery, the patients were rinsed with 0.1% chlorhexidine mouthwash (ICPA Health Products, Ankleshwar, India) for one minute, and the perioral mucosa was disinfected with povidone-iodine solution (PSK Pharma Pvt. Ltd., Karnataka, India). Local anesthesia (Lignox 2% with 1:80,000 adrenaline, Indoco Remedies Limited, Mumbai, India) was administered at the implant site. Soft tissue parameters, including mucosal thickness and BOP, were evaluated preoperatively. Mucosal thickness was measured using Endodontic K-file No. 20 (MANI, Utsunomiya, Japan) with a rubber stopper inserted at the midpoint of the attached gingiva between the mucogingival junction and an imaginary line connecting the cementoenamel junction (CEJ) of adjacent teeth. The distance from the file tip to the rubber stopper was measured using a digital caliper (Baker Gauges; Kwatra Enterprises, Delhi, India) to the nearest 0.01 mm. BOP was assessed using a pressure-sensitive probe (GDC, Hoshiarpur, India) with a force of 0.25 N/cm, passed along the gingival sulcus. After 30 s, bleeding was scored using the Modified Sulcular Bleeding Index by Mühlemann and Son [10]: zero (no bleeding), one (pinpoint bleeding), two (thin linear rim of bleeding), or three (profuse bleeding).

Following anesthesia, a crestal incision was made with a No. 15 BP blade, and a full-thickness mucoperiosteal flap was reflected. The surgical stent was seated, and a 2 mm pilot drill (AlphaBio, Petach Tikva, Israel) marked the implant site. Parallelism was verified using a parallel pin, followed by sequential drilling with a physio-dispenser (W&H, Dentalwerk, Burmoos, Austria) under internal and external saline irrigation (sodium chloride injection IP; Abaris Healthcare Pvt. Ltd., Raipur, India). Angulation of the osteotomy site was checked using a paralleling pin to ensure accuracy. Two-piece titanium dental implants (AlphaBio ICE, Petach Tikva, Israel) with dimensions selected based on CBCT evaluation were inserted until the implant head was at the level of the alveolar crest. Implant stability was measured using resonance frequency analysis (RFA) with an Osstell ISQ device (Osstell AB, Gamlestadsvagen, Göteborg, Sweden). A SmartPeg was attached to the implant, and stability was recorded in the buccal, lingual, mesial, and distal directions, with the average Implant Stability Quotient (ISQ) noted. The SmartPeg was removed, a cover screw was placed, and the flaps were approximated using 3-0 non-absorbable silk sutures (Mersilk, Johnson & Johnson Pvt. Ltd., Aurangabad, India).

**Figure 1:**
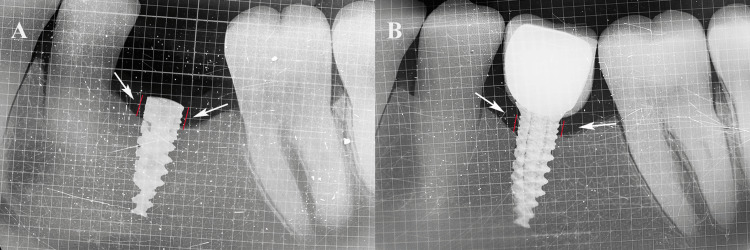
Radiograph findings. Marginal bone loss was measured using 1-mm grid–enabled intraoral periapical radiographs (IOPAs) (A) after three months, at the time of the second surgical procedure, and (B) after six months, post-loading. Red line denotes the distance between the implant shoulder and alveolar crest on mesial and distal sides as marginal bone loss in mm (white arrows). Original IOPAs of the patient from the study were used with the patient's permission.

The marginal bone level was assessed using an intraoral periapical (IOPA) radiograph taken with a paralleling technique utilizing a customized occlusal bite jig made from modeling wax (Maarc, Mumbai, India) attached to a film holder (Rinn XCP, Dentsply, York, PA, USA), which was later acrylized in heat-cure acrylic (DPI, Uttarakhand, India) for standardization. The radiograph included a 1 mm grid, and the distance from the implant shoulder to the alveolar crest at the mesial and distal bone-implant contact points was measured (Figure 1).

**Figure 2:**
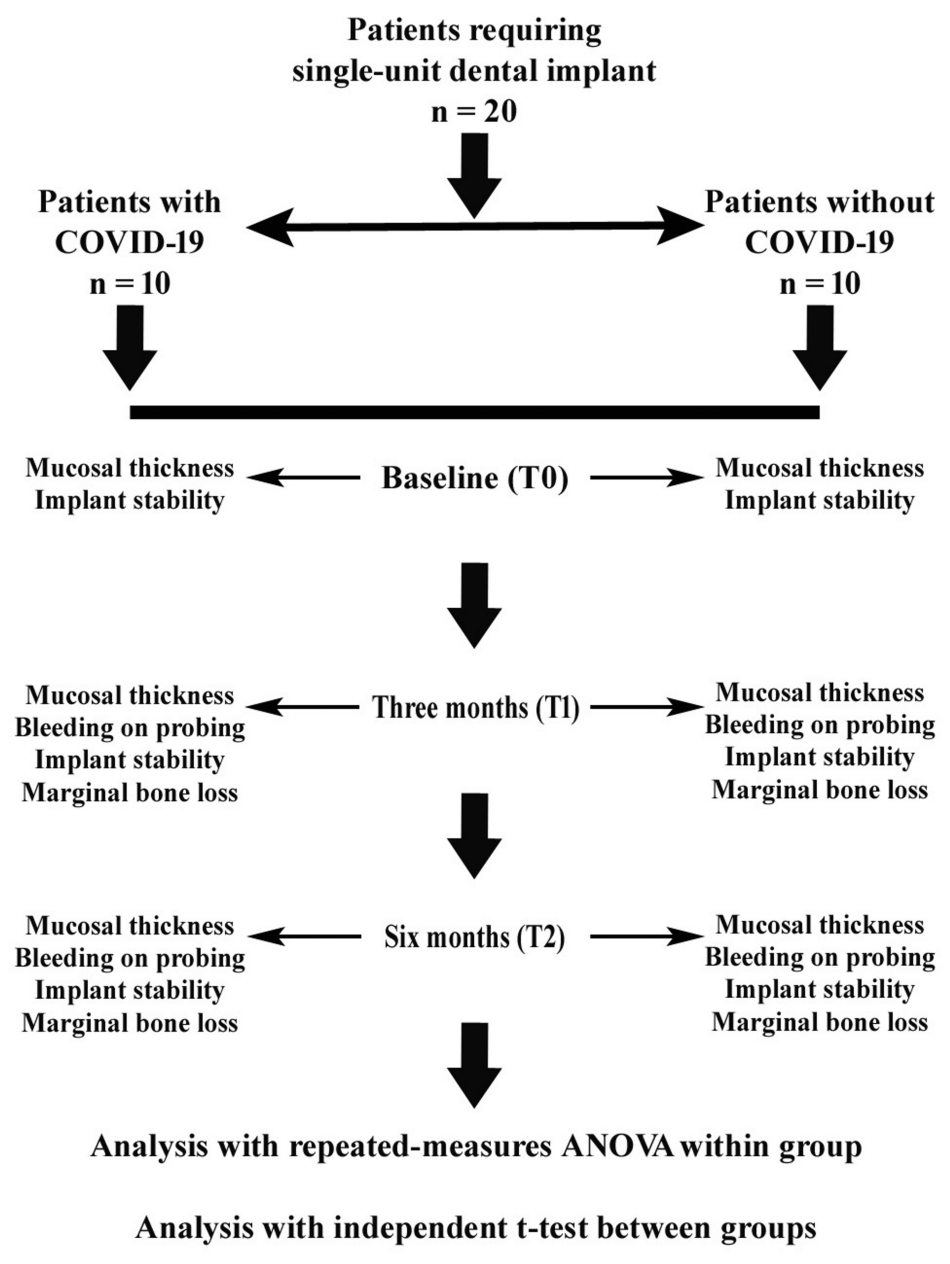
Study flowchart. ANOVA: analysis of variance; COVID-19: coronavirus disease 2019

Postoperative medications (amoxicillin 500 mg, three times daily for five days; ibuprofen 400 mg, as needed; Alkem Laboratories, Mumbai, India) and instructions were provided, and the sutures were removed after one week. At the three-month follow-up, the patient underwent a second-stage surgery for definitive prosthesis fabrication. Soft tissue parameters (mucosal thickness and BOP) were evaluated using the same methods. The marginal bone level was assessed using IOPA radiography and compared with the baseline to calculate marginal bone loss. Implant stability was measured using the RFA. The implant was exposed, the cover screw was replaced with a healing cap, and the site was left for one week to allow gingival contouring. A closed-tray impression was made using an elastomeric impression material (3M ESPE, St. Paul, MN, USA) in a stock tray (Jabbar Company, Lahore, Pakistan). The impression coping was attached to an implant analog, and the cast was poured with a type IV gypsum product (Zooenta; Neelkanth Healthcare Pvt. Ltd., Jodhpur, India). A screw-retained metal-ceramic crown was fabricated, and during insertion, the abutment screw access hole was packed with Teflon tape and sealed with composite material (3M ESPE, St. Paul, MN, USA). Fit and occlusion were verified using the conventional protocols. At the post-loading six-month follow-up, all parameters (mucosal thickness, BOP, implant stability, and marginal bone loss) were re-evaluated using the same standardized methods. Implant stability was measured using RFA, and the prosthesis was reinserted after evaluation. The study flowchart is shown in Figure 2.

All the measurements were performed by a single calibrated examiner to ensure reliability. Calibration was conducted by measuring mucosal thickness, BOP, and implant stability in five non-study patients twice, with a 48 h interval, achieving an intra-examiner reliability of ≥ 0.85 (Cohen’s kappa for BOP; intraclass correlation coefficient for continuous variables). Radiographic measurements were standardized using a customized bite jig to ensure consistent film placement and cone angulation. The data collected at baseline and at three and six months were subjected to statistical analyses.

Statistical analysis was performed using IBM SPSS Statistics for Windows, Version 25 (Released 2017; IBM Corp., Armonk, New York, United States). The Shapiro-Wilk test (p > 0.05) confirmed normal data distribution. For within-group comparisons across multiple time points, repeated-measures analysis of variance (ANOVA) was applied, followed by post-hoc Bonferroni correction. Independent t-tests were used for between-group comparisons of outcome variables. All tests were two-tailed, and statistical significance was set at α = 0.05. Data are expressed as the mean ± standard deviation (SD).

## Results

The demographic characteristics of the patients are presented in Table 1. The mean age of non-COVID-19 patients was 34.25 ± 4.56 years, while COVID-19 patients had a slightly lower mean age (32.70 ± 5.85 years). The overall mean age across both groups was 34.10 ± 4.15 years, suggesting a relatively similar age distribution. Sex distribution was equal, with each group consisting of six (30%) males and four (20%) females, totaling 12 (60%) males and eight (40%) females in the overall sample. The total number of patients was evenly distributed, with 10 (50%) in each group, totaling 20 (100%). The results indicated no substantial demographic differences between groups.

**Table 1:** Demographic characteristics of the sample. Data are presented as mean and standard deviation (SD), whereas sex is presented as frequency (n) and percentage (%), where n denotes the number of patients. COVID-19: coronavirus disease 2019

Variables		Non-COVID-19	COVID-19	Overall
Age (years)	Mean ± SD	34.25 ± 4.56	32.70 ± 5.85	34.10 ± 4.15
Male	n (%)	6 (30)	6 (30)	12 (60)
Female	n (%)	4 (20)	4 (20)	8 (40)
Total	n (%)	10 (50)	10 (50)	20 (100)

Repeated-measures ANOVA with Bonferroni post-hoc analysis in non-COVID-19 patients revealed significant changes in the parameters over time. Dental implant stability showed no significant variation (p = 0.634), and pairwise comparisons were not significant (p > 0.05). In contrast, marginal bone loss exhibited a significant increase (p = 0.001), with T0 differing markedly from T1 (mean difference = -0.49 mm, p = 0.001) and T2 (mean difference = -0.57 mm, p = 0.001), although T1 vs. T2 was not significant (p = 0.053). Bleeding scores showed a marginal time effect (p = 0.03) but no significant pairwise differences. Mucosal thickness was significantly decreased by T2 (p = 0.001), with notable reductions between T0-T2 (mean difference = 0.35 mm, p = 0.001) and T1-T2 (mean difference = 0.40 mm, p = 0.002). These findings suggested that while implant stability remained consistent, progressive bone loss occurred post-treatment, warranting further monitoring. Late-phase mucosal thinning might reflect tissue adaptation, whereas bleeding trends required further investigation because of their marginal significance (Table 2).

**Table 2:** Analysis of outcome parameters at multiple time intervals using repeated-measures analysis of variance (ANOVA) test followed by post-hoc Bonferroni test in non-COVID-19 patients. F-statistics were calculated using repeated-measures analysis of variance (ANOVA), and t-statistics were calculated by post-hoc Bonferroni test. *p < 0.05 denotes statistical significance. Data are presented as mean and standard deviation (SD). COVID-19: coronavirus disease 2019; ISQ: Implant Stability Quotient; T0: baseline; T1: three months after implant placement at second stage surgery; T2: six months post-loading

Parameters	Time intervals	Non-COVID-19		F-statistics	p-value	Pairwise comparison	Mean difference	t-statistics	p-value
		Mean	SD						
Implant stability (ISQ)	T0	75.47	8.88	0.46	0.634	T0 vs T1	2.85	0.62	0.999
	T1	72.62	9.58			T0 vs T2	2.75	1.12	0.860
	T2	72.72	7.98			T1 vs T2	-0.10	-0.03	0.999
Marginal bone loss (mm)	T0	0	0	29.79	0.001*	T0 vs T1	-0.49	-5.60	0.001*
	T1	0.49	0.27			T0 vs T2	-0.57	-5.60	0.001*
	T2	0.57	0.32			T1 vs T2	-0.08	-2.20	0.053
Bleeding on probing (scores)	T0	0	0	4.23	0.030*	T0 vs T1	-0.40	-2.40	0.110
	T1	0.40	0.51			T0 vs T2	-0.40	-2.40	0.110
	T2	0.40	0.51			T1 vs T2	0	0	1
Mucosal thickness (mm)	T0	2.88	0.53	16.80	0.001*	T0 vs T1	-0.05	-0.61	0.555
	T1	2.93	0.58			T0 vs T2	0.35	6.32	0.001*
	T2	2.53	0.44			T1 vs T2	0.40	4.71	0.002*

Longitudinal analysis of COVID-19 patients revealed distinct patterns in peri-implant parameters. While implant stability showed a non-significant time effect (p = 0.07), pairwise comparisons suggested a marginal improvement from T1 to T2 (p = 0.06). Bone loss demonstrated significant progression (p < 0.001) with substantial increases at all time points (p < 0.001). The bleeding scores remained stable without significant temporal changes (p = 0.276). Mucosal thickness showed a progressive reduction (p < 0.001), with a significant decrease between all intervals (p < 0.05). These findings suggest that COVID-19 patients experience accelerated peri-implant bone loss compared to their reported non-COVID-19 counterparts, potentially reflecting systemic inflammatory impacts. Progressive mucosal thinning, in contrast to the pattern observed in non-COVID-19 patients, may indicate distinct healing responses. The borderline stability changes (T1-T2) may represent delayed osseointegration (Table 3).

**Table 3:** Analysis of outcome parameters at multiple time intervals using repeated-measures analysis of variance (ANOVA) test followed by post-hoc Bonferroni test in COVID-19 patients. F-statistics were calculated using repeated-measures analysis of variance (ANOVA), and t-statistics were calculated by post-hoc Bonferroni test. *p < 0.05 denotes statistical significance. Data are presented as mean and standard deviation (SD). COVID-19: coronavirus disease 2019; ISQ: Implant Stability Quotient; T0: baseline; T1: three months after implant placement at second stage surgery; T2: six months post-loading

Parameters	Time intervals	COVID-19		F-value	p-value	Pairwise comparison	Mean difference	t-value	p-value
		Mean	SD						
Implant stability (ISQ)	T0	70.27	12.31	2.94	0.070	T0 vs T1	8.40	2.10	0.190
	T1	61.87	10.06			T0 vs T2	4.16	0.65	0.999
	T2	67.55	8.93			T1 vs T2	2.06	-2.74	0.060
Marginal bone loss (mm)	T0	0	0	45.25	0.001*	T0 vs T1	-0.61	-6.68	0.001*
	T1	0.61	0.28			T0 vs T2	0.90	-7.01	0.001*
	T2	0.90	0.40			T1 vs T2	-0.29	-5.11	0.001*
Bleeding on probing (scores)	T0	0	0	1.38	0.276	T0 vs T1	-0.20	-1.50	0.540
	T1	0.20	0.42			T0 vs T2	-0.20	-1.50	0.540
	T2	0.20	0.42			T1 vs T2	0	0	1
Mucosal thickness (mm)	T0	2.37	0.71	49.55	0.001*	T0 vs T1	0.22	9.00	0.001*
	T1	2.15	0.68			T0 vs T2	0.33	9.00	0.001*
	T2	2.03	0.73			T1 vs T2	0.11	2.80	0.019*

A comparative analysis revealed significant differences between the COVID-19 and non-COVID-19 groups. Implant stability was significantly lower in COVID-19 patients at T1 (p = 0.02), although non-significant at T0/T2. Marginal bone loss showed marginal significance at T2 (p = 0.05), suggesting accelerated bone resorption in COVID-19 patients over time. The bleeding scores showed no significant differences between the groups. Mucosal thickness was significantly reduced in COVID-19 patients at T1 (p = 0.01), indicating altered healing patterns. These findings suggested COVID-19 patients might experience delayed osseointegration (T1 stability) and greater late-stage bone loss, potentially due to systemic inflammatory effects. The transient mucosal thickness differences implied COVID-19-specific healing responses, warranting closer monitoring of post-COVID-19 implant cases (Table 4).

**Table 4:** Comparison for outcome parameters between the COVID-19 and non-COVID-19 groups at multiple time intervals with an independent t-test. *p < 0.05 denotes statistical significance using an independent t-test, mean difference: non-COVID-19 group - COVID-19 group. COVID-19: coronavirus disease 2019; ISQ: Implant Stability Quotient; T0: baseline; T1: three months after implant placement at second stage surgery; T2: six months post-loading.

Parameters	Time intervals	Mean difference of groups	t-value	p-value
Implant stability (ISQ)	T0	5.20	-1.08	0.29
	T1	10.75	-2.44	0.02*
	T2	5.17	-1.36	0.18
Marginal bone loss (mm)	T0	0	0	1
	T1	-0.12	0.94	0.35
	T2	-0.33	2.02	0.05
Bleeding on probing (scores)	T0	0	0	1
	T1	0.20	-0.94	0.35
	T2	0.20	-0.94	0.35
Mucosal thickness (mm)	T0	0.51	-1.82	0.08
	T1	0.78	-2.75	0.01*
	T2	0.50	-1.83	0.08

## Discussion

The results of this study revealed significant differences in peri-implant parameters, particularly marginal bone loss and mucosal thickness, suggesting that a history of COVID-19 may influence implant success through systemic inflammatory and metabolic pathways. Implant stability remained relatively consistent in both groups, with no significant variation over time in non-COVID-19 patients and borderline improvement in COVID-19 patients from T1 to T2. This stability aligns with the findings of Becker et al. [11], who reported that RFA values typically stabilize post-osseointegration, reflecting successful implant integration, and that these values increase over time. However, the marginal improvement in COVID-19 patients contrasts with the findings of Block MS [7], suggesting delayed osseointegration due to systemic inflammation. For instance, Block MS [7] reported reduced early stability in post-COVID-19 patients, which was attributed to altered bone remodeling from cytokine storms. The borderline significance in this study may indicate a delayed healing response, potentially linked to residual inflammatory mediators such as interleukin-6, which are elevated post-COVID-19 and can impair osteoblast activity [5].

A key finding was the significant increase in marginal bone loss over time, particularly in COVID-19 patients, with notable differences compared to non-COVID-19 patients at T2. This accelerated bone resorption supports the hypothesis that COVID-19 affects bone metabolism, possibly through mechanisms involving receptor activator of nuclear factor κB ligand/osteoprotegerin (RANKL/OPG) dysregulation [5]. A previous study demonstrated that SARS-CoV-2 infection led to an increase in the RANKL/OPG ratio owing to a decrease in OPG, inducing osteoclast activation and leading to enhanced bone resorption [12]. Moreover, markers indicative of bone turnover may exhibit alterations, as evidenced by a study that demonstrated that serum concentrations of the C-terminal telopeptide of type 1 collagen (CTX), a peptide associated with bone resorption, and osteocalcin, a biomarker for bone formation, were diminished in patients infected with SARS-CoV-2 compared to age- and sex-matched controls who were uninfected with SARS-CoV-2, suggesting a decrease in bone turnover [13].

In contrast, Sezer et al. [14] found no association between COVID-19 infection and early implant failure, suggesting that the effect may depend on the severity of the infection or individual immune responses. The progressive bone loss in the COVID-19 group in this study may reflect a prolonged inflammatory state, as COVID-19 is known to disrupt calcium homeostasis and vitamin D metabolism, both of which are critical for bone integrity [4]. Bleeding scores showed a marginal time effect in non-COVID-19 patients but remained stable in COVID-19 patients, with no significant between-group differences. This stability contrasts with the expectation of heightened inflammation in post-COVID-19 patients, where endothelial dysfunction may increase bleeding tendencies. The lack of significant change here may indicate effective postoperative management or that the six-month follow-up was insufficient to capture late inflammatory effects. The marginal significance in non-COVID-19 patients may reflect natural peri-implant tissue adaptation, rather than pathology. AlAhmari et al. [9] reported no short-term adverse effects of acute COVID-19 on peri-implant tissues.

Mucosal thickness significantly decreased over time in both groups, with distinct patterns: progressive reduction in COVID-19 patients and late-phase thinning in non-COVID-19 patients. The significant reduction in COVID-19 patients at T1 suggests an altered healing response, potentially due to fibrosis or reduced collagen synthesis following systemic inflammation [15]. Sahin and Akkus [16] found that COVID-19-induced fibroblast dysfunction leads to thinner mucosal tissue. In contrast, non-COVID-19 patients showed delayed thinning, possibly reflecting normal tissue remodeling post-surgery. This difference highlights a COVID-19-specific impact, possibly mediated by TGF-β dysregulation, which is implicated in fibrotic changes after viral infection [17].

The observed results can be linked to COVID-19’s systemic effects on bone and soft tissue metabolism [5]. SARS-CoV-2 infection triggers a cytokine storm, releasing pro-inflammatory cytokines (such as IL-1, IL-6, and TNF-α) that disrupt the osteoblast-osteoclast balance, favoring bone resorption [5,18]. This is supported by elevated C-reactive protein levels in post-COVID-19 patients, which correlate with bone loss [4]. Additionally, COVID-19-related hypocalcemia and vitamin D deficiency impair mineralization and exacerbate bone turnover [19]. In soft tissues, endothelial damage and microvascular thrombosis [20] may reduce mucosal thickness by impairing the nutrient supply and promoting fibrosis. These mechanisms likely explain the accelerated bone loss and altered mucosal dynamics in COVID-19 patients.

Clinical implications

These findings have significant implications for implant dentistry. Clinicians should consider a patient’s COVID-19 history during treatment planning, potentially extending healing periods or enhancing bone augmentation in post-COVID-19 cases to mitigate bone loss. Monitoring mucosal thickness and stability at early intervals can guide interventions such as soft-tissue grafting. Prophylactic anti-inflammatory therapies or vitamin D supplementation should be explored, although further research is required. Patient education during long-term follow-up is crucial for detecting progressive changes.

Limitations

The study’s small sample size limits generalizability, and the six-month follow-up may not capture the long-term outcomes. Variability in COVID-19 severity and time since infection was not controlled for, potentially confounding the results. While calibrated, single-examiner measurements may have introduced bias. Another limitation was the absence of biochemical markers to directly link COVID-19 to inflammatory bone resorption. To validate these findings, future studies should include larger cohorts, longer follow-up periods, and stratification according to infection severity.

## Conclusions

In conclusion, this study demonstrated that a history of COVID-19 significantly impacted peri-implant outcomes, with COVID-19-affected patients exhibiting accelerated marginal bone loss and progressive mucosal thinning compared to non-COVID-19 patients over a six-month period. The marginal improvement in implant stability in the COVID-19 group and the distinct healing patterns in soft tissue parameters suggested delayed osseointegration and altered tissue remodeling, likely driven by systemic inflammatory effects. These findings underscore the necessity for clinicians to adjust treatment protocols, such as extending healing periods and monitoring bone and soft tissue changes more closely in post-COVID-19 patients, to enhance implant success rates. Further longitudinal studies with larger cohorts are warranted to validate these observations and refine therapeutic strategies.
